# The Study of Security Priming on Avoidant Attentional Biases: Combining Microsaccadic Eye-Movement Measurement With a Dot-Probe Task

**DOI:** 10.3389/fpsyg.2021.726817

**Published:** 2021-10-21

**Authors:** Rebecca Louise Mellor, Elia Psouni

**Affiliations:** Department of Psychology, Lund University, Lund, Sweden

**Keywords:** attentional biases, eye tracking, microsaccades, dot-probe design, attachment orientation, security priming, avoidance

## Abstract

Microsaccades are small fixational eye movements that have shown to index covert attentional shifts. The present experiment combined microsaccades with performance measures from a dot-probe task to study influences of attachment security priming on the attentional biases of individuals high in attachment avoidance. Security priming is an experimental manipulation aimed at boosting felt security. Using a randomized, mixed design, we measured differences in attentional vigilance toward angry and neutral faces as a function of priming (neutral vs. secure) and attachment avoidance. Individuals high in avoidance habitually tend to withdraw from, or otherwise dismiss, emotionally salient stimuli. Here, we operationalized attentional withdrawal based on both task performance in the dot-probe task and microsaccadic movements. In addition, unlike previous studies where priming salience for the individual participant has been unclear, we used a standardized narrative method for attachment script assessment, securing an indication of how strongly each participant was primed. Dot-probe data significantly captured the link between avoidance and attentional disengagement, though from *all* facial stimuli (angry and neutral). Although microsaccadic movements did not capture avoidant attentional disengagement, they positively correlated to dot-probe data suggesting measurement convergence. Avoidance was associated with weaker security priming and no overall effect of priming on attention was found, indicating a need for further exploration of suitable priming methods to bypass avoidant deactivation. Our results provide a first indication that, as an implicit looking measure, microsaccadic movements can potentially reveal where early attention is directed at the exact moment of stimulus presentation.

## Introduction

### Cognitive Biases Associated With Attachment Avoidance

Attachment theory posits that early childhood experiences with caregivers result in internal working models that shape interpersonal relationships in adulthood (Bowlby, 1969). Attachment in adulthood is often conceptualized along dimensions “anxiety” and “avoidance.” Low scores on both denote secure attachment, while high scores on one or the other denote insecure attachment (Bartholomew and Horowitz, 1991). Insecure-anxious attachment manifests as fear of abandonment and rejection in relationships, while insecure-avoidant attachment is associated with discomfort with relational closeness and intimacy, and emotional distance. Positive links have been shown between attachment security and adaptive coping, affect regulation and psychological resilience (Bartholomew and Horowitz, 1991; Mikulincer and Shaver, 2001), sophisticated executive functions, and academic performance (van IJzendoorn et al., 1995; Aviezer et al., 2002). Conversely, insecure attachment styles are construed as less adaptive and are linked to typical ways of processing emotional and relational information, distinctively different for attachment avoidance and attachment anxiety (Dykas and Cassidy, 2011). Accordingly, research has focused on understanding the underlying cognitive mechanisms for these information processing patterns. It has been suggested that specifically avoidant attachment is linked to early attentional biases to avoid emotional stimuli. Since attachment avoidance is linked to several negative outcomes, e.g., loneliness, depression, anxiety (e.g., McWilliams and Bailey, 2010), and substance use disorder (e.g., Caspers et al., 2005), a better comprehension of the associated lower-level cognitive mechanisms is warranted.

Research suggests that individuals with avoidant attachment exhibit impaired social and emotional information processing. Most established are impairments in attention to, and memory of, attachment-related stimuli in long-term memory (Fraley et al., 2000a; Edelstein, 2006) and working memory (Edelstein, 2006), and impairments in facial emotion recognition (Escobar et al., 2013). These impairments are allegedly partly caused by early attentional processing biases, what Bowlby (1980) termed defensive exclusion. Thus, in individuals with avoidant attachment, attention is diverted away from positive and negative attachment-related stimuli, such as attachment-related words, pictures, and emotional faces (e.g., Fraley et al., 2000a; Dewitte et al., 2007; Dewitte and De Houwer, 2008; Vrticka et al., 2008; Dewitte, 2011; Vrticka and Vuilleumier, 2012; Winer and Salem, 2016). Importantly, avoidant attentional biases express as attentional hypervigilance initially (at 100ms), and disengagement from the stimulus later (Fraley et al., 2000a), at 750–1000ms (Chun et al., 2015; Winer and Salem, 2016).

### Security Priming as Moderator of Avoidant Strategies

Security priming, an experimental manipulation that alters felt security by activating relevant mental representations, can moderate negative outcomes linked with insecure attachment (Gillath and Karantzas, 2019). For instance, security priming has been shown to reduce aggression (Mikulincer and Shaver, 2007), increase creative problem-solving capacity (Mikulincer et al., 2011), and reduce stress over time (Oehler and Psouni, 2018). Priming also influences memory processing, enhancing recall of positive attachment-related words, and relational expectations (Rowe and Carnelley, 2003). This evidence on benefits of security priming warrants a better understanding of the mechanisms by which it may exert its influence.

Security priming often involves exposure to symbolic pictures or security-related words, and, depending on the time length of exposure, can be subliminal or supraliminal. Research indicates that subliminal priming may not be salient enough to elicit felt security (Andriopoulos and Kafetsios, 2015). Supraliminal priming may be more efficient in activating recalled instances of felt support but when explicitly personal, it may fail to activate attachment information in avoidant individuals with little access to, and poorer recall of, attachment-related interactions. Evidently, while security priming seems to have positive effects regardless of attachment orientation, some findings suggest that avoidant individuals are less prone to this influence (Carnelley and Rowe, 2007; Bryant and Chan, 2017; Gillath and Karantzas, 2019). Thus, when looking at potential effects of security priming on avoidant biases, a measure of the strength of the prime appears necessary to directly assess how strongly each individual is primed.

### Microsaccades as Visual Attention Measure in Dot-Probe Designs

Dot-probe designs with emotionally expressive facial stimuli are often used for studying avoidant attentional biases. In these designs, avoidance is associated with slower responses to dots presented on the same side as emotionally expressive faces (congruent trials), and faster responses to dots presented on the opposite side of expressive faces (incongruent trials; Dewitte et al., 2007; Edelstein and Gillath, 2008; Chun et al., 2015), taken to indicate attention disengagement (decreased vigilance) from the emotional stimulus.

Dot-probe designs often involve simultaneous presentation of an emotional and a neutral stimulus, and attentional orientation indexes (AOIs) that indicate the difference in reaction times (RTs) in congruent versus incongruent trials (MacLeod et al., 1986). However, AOIs cannot distinguish between vigilance to one stimulus from difficulty to disengage from another (Fox et al., 2001; van Rooijen et al., 2017), making designs presenting one stimulus preferable (Chun et al., 2015). Another concern is the extent to which RTs can support inferences about cognition (Staugaard, 2009; van Rooijen et al., 2017), as they only capture a snapshot of attentional allocation, at target detection (Cooper and Langton, 2006; Andriopoulos and Kafetsios, 2015), not differentiating between overt and covert attention (Petrova et al., 2013).

These issues may be addressed by tracking eye movements during dot-probe tasks. Indeed, overt eye movements correlate with AOIs (Mogg et al., 2007; Petrova et al., 2013; Price et al., 2015), but attachment-related attentional biases have not been investigated with eye tracking. Notably, these biases entail covert attentional shifts of focus that may not demonstrate in overt eye movements. Furthermore, eye-tracking studies use proportion or length of first fixations on emotional, compared to neutral, stimuli, but these indices may be reliable only for longer stimulus presentations (5000ms; Waechter et al., 2014). This is particularly problematic when studying avoidant biases in early attentio 500ms).

We suggest that microsaccades may be more appropriate for assessing early, covert attentional biases. Microsaccades, alongside tremor and drift, are very small eye movements that help counteract retinal adaptation, maintaining our ability to view stationary scenes (Engbert and Kliegl, 2003). They occur typically 1–2 times/s with amplitudes <1 degree of the visual angle, and, unlike tremor and drift, can index the orientation of covert attentional shifts (Hafed and Clark, 2002; Engbert and Kliegl, 2003; Hafed et al., 2013; Yuval-Greenberg et al., 2014). Microsaccades, which occur quickly upon stimulus presentation, cease at around 150ms, and occur again maximally at around 350ms before returning to baseline, can be detected based on velocity thresholds (Engbert and Kliegl, 2003). Numerous replications (e.g., Rolfs et al., 2004, 2006; Laubrock et al., 2005) ascertain that the directional distribution of microsaccades can index reflexive shifts in attention during prolonged fixations. Since microsaccades are not consciously controlled, they may be useful as indicators of early attention allocation in dot-probe designs.

Thus, along with RT-data gained from a dot-probe design, we used microsaccadic movements for indexing attentional biases associated with attachment avoidance. Angry faces were used as stimuli, as they convey negative interpersonal information and produce heightened arousal (Lundqvist et al., 2015; van Rooijen et al., 2017), activating avoidant disengagement strategies (Dewitte, 2011). We expected microsaccadic movement data to correlate with AOIs in the dot-probe task. For priming, we used a standardized task that activates attachment knowledge without being overtly personal, and which renders a quantification of attachment security activated by each participant. As the focus was on attachment avoidance, we ensured through pre-screening that participants represented either avoidant or non-avoidant attachment (secure), with similar (low) levels of anxiety.

H1: Overall, there will be more microsaccadic eye movements away from angry faces, compared to neutral faces, thereby capturing avoidant disengagement on the visual behavior level.

H2: High avoidance will predict lower vigilance toward angry faces compared to neutral faces pre-priming, in line with evidence of characteristic avoidant attentional biases.

H3: Priming will influence attention to facial expressions of low avoidance but not high avoidance participants, given evidence that priming may not affect avoidant attentional biases.

## Materials and Methods

### Participants

Participants (*N*=40, 12 male/28 female) were 21–39years old (*M*=27.40, *SD*=4.47) and had normal or corrected-to-normal vision. They were recruited after pre-screening to have moderate-to-low-attachment anxiety, with score-range 1.22–3.94 (*M*=2.93, *SD*=0.71). Avoidance scores ranged from 1.22 to 5.72 (*M*=2.89, *SD*=1.04).

### Materials

#### Experiences in Close Relationships Revised

We measured attachment at pre-screening with Experiences in Close Relationships Revised (ECR-R; Fraley et al., 2000b), a 36-item self-report assessing attachment style along the dimensions of anxiety and avoidance with high reliability (based on previous data *α*=0.94 for Avoidance and *α*=0.91 for Anxiety; Brennan et al., 1998 and in the present study, Anxiety *α*= 0.80 and Avoidance *α*= 0.80).

#### Priming Tasks

We randomly assigned participants to either secure or neutral priming. In both conditions, participants created two stories using twelve-word prompts to guide each story (Supplementary Material 1.1). Stories in the secure priming condition came from the Secure-Base Script Test (SBST: Psouni and Apetroaia, 2014), aimed at activating secure-base scripts. Stories in the neutral condition were modified from Attachment Script Assessment (ASA; Waters and Rodrigues-Doolabh, 2004) and intended to elicit neutral information. Stories were scored for evidence of attachment security scripts, based on a coding manual (Psouni and Apetroaia, 2013), by two reliable coders (intra-class and inter-coder reliability=0.89). No neutral priming story contained any secure-base script features.

#### Attentional Orientation Task

A dot-probe task was used to assess attentional orientation, presenting an angry or neutral face followed by a visual probe (dot) on one side of the screen. Attentional shifts away/toward the face were measured by RTs to dots in congruent versus incongruent trials. Each trial started with a 0.035 degree black fixation cross at the center of the screen, presented first alone (1000ms), then together with a face (1000ms), total 2000ms. The 000ms face presentation enabled capturing avoidant disengagement, which occurs most reliably around 750–1000ms after stimulus presentation (Winer and Salem, 2016). As microsaccades occur 1–2 times/1000ms, this was also appropriate for capturing microsaccadic activity within a timeframe corresponding to early attention. As elsewhere (Petrova et al., 2013; Chun et al., 2015), participants were instructed to keep looking at the fixation cross until the dot appeared at 2000ms, set to display for 2000ms or until the participant response indicating its position (pressing “Q” for left or “P” for right). A randomized time length pause was used between trials (800ms-1200ms) to ensure that naturally occurring microsaccade cycles did not confound recordings.

The facial stimuli, taken from Radboud Faces Database after permission (Langner et al., 2010), consisted of eight angry and eight neutral faces (counterbalanced gender). There was a 50% probability of each face to appear on each side of the screen. A total of 75% trials were congruent (96 trials per block and 48 angry/48 neutral) to maintain the expectancy that cues typically predict target location. Reliability (Cronbach’s *α*) here for angry faces was *α*= 0.85 for incongruent trials and *α*= 0.82 for congruent trials. Reliability for neutral faces was *α*= 0.86 for incongruent trials and *α*= 0.81 for congruent trials – together suggesting high internal consistency in the dot-probe task in the present study. See Supplementary Material 1.2 for one trial.

#### Eye Tracking

Binocular eye movements were tracked using a Tobii Spectrum screen-based tracker. The sampling rate was 600Hz. Viewing distance was constant (60cm from the screen) and head movements kept to a minimum using a forehead-and-chin rest throughout. A standardized calibration procedure preceded the experiment (proponent of Titta: Niehorster et al., 2020): Participants fixated on nine dots appearing in a systematic pattern. Values >1 were satisfactory. Re-calibration was performed if visual inspection revealed points with large deviations, or if the values were<1. Calibration accuracy rates (left/right eye) were satisfactory. Microsaccades were detected using a standard algorithm (Engbert and Kliegl, 2003).

#### Positive and Negative Affect Scale

Positive and Negative Affect Scale (PANAS; Watson et al., 1988) was used for assessing the impact of priming on mood. Participants rate adjectives, such as “interested” or “irritable” for consistency/inconsistency with their current mood. Reported reliability ranges 0.86–0.90 for positive and 0.84–0.87 for negative affect (Watson et al., 1988) and was here for both the positive and negative subscale *α*= 0.85 pre-priming and *α*= 0.85 post-priming.

#### State–Trait Anxiety Inventory

State–Trait Anxiety Inventory (STAI; Spielberger and Gorsuch, 1983) was used as control measure for anxiety. Items represent anxious feelings in the present moment (20 items) and by disposition (20 items). Internal consistency ranges 0.81–0.95 (McDowell, 2006) and was in the present study *α*= 0.93 for both State and Trait Anxiety.

### Procedure

Recruitment to the pre-screen was through announcement on social media platforms. The ECR-R was filled out on-line and only individuals with anxiety scores <3.96 were invited to participate. This cutoff was ascertained through averaging anxiety scores from seven studies on the psychometric properties of the ECR-R (Tsagarakis et al., 2007; Alonso-Arbiol et al., 2008; Olssøn et al., 2010; Holland et al., 2012; Busonera et al., 2014; Esbjørn et al., 2015). To ensure no inadvertent priming of attachment-related thoughts from the ECR-R (Cassidy et al., 2009; Chun et al., 2015), 10days were allowed between pre-screen and experiment. Overall, 102 adults (35 males/67 females) 20–36years old filled the pre-screen, and 80 met the inclusion criterion and were invited to the experiment. Those who participated (*N*=40) had higher attachment anxiety scores than those who did not (*p*=0.010). The two groups did not differ on avoidance, age, or gender.

The experiment took place at X and was computer-based, using PsychoPy software. Stimuli and instructions were presented in black text on a mid-gray background. After providing consent, participants completed PANAS and State/STAI, and carried out 10 practice dot-probe trials (only neutral faces), followed by a pre-priming block (128 trials). The priming task was then carried out, taking 10min. Stories were recorded and later transcribed and scored. Then, PANAS was repeated, followed by the post-priming dot-probe block (128 trials), and the Trait/STAI. Participants were finally debriefed and compensated.

### Data Preparation

Pre-processing of dot-probe data included removing trials with incorrect responses and RTs shorter than 150ms or exceeding 2000ms (less than 1%). Outlying RTs were Winsorized to the lower and higher RT point within the +/−2 SD range (1.88% of RT-data). For AOIs, mean RT on congruent trials was subtracted from mean RT on incongruent trials (Andriopoulos and Kafetsios, 2015; Chun et al., 2015):


$$\begin{array}{l} AttentionalOrientation=RTincongruenttrials\\ \;-RTcongruenttrials. \end{array}$$

Four attentional orientation indexes were calculated per participant, one for each stimulus type (angry pre-priming, neutral pre-priming, angry post-priming, and neutral post-priming). A positive AOI value indicates that participants responded faster to congruent, compared to incongruent, trials, denoting sustained concentration toward the stimulus face (attentional vigilance). Conversely, negative AOI values indicate that participants responded faster to incongruent compared to congruent trials, denoting attentional disengagement from the stimulus facial expression.

Raw eye-movement data were gained through the Titta-toolbox (Niehorster et al., 2020), which integrates the Tobii eye tracker with experimental software (PsychoPy; Peirce et al., 2019). Only data obtained in the facial stimulus presentation window (1000ms) were analyzed. Only binocular microsaccades were considered. Microsaccades were detected using the Engbert and Kliegl (2003) algorithm with parameters *λ*=6. The peak microsaccade velocity was ascertained through a data driven threshold using the SD of the velocity components. Minimum saccade duration was 6ms while amplitudes were less than 1 degree. Time points with missing data were removed from the analysis. Data were also filtered for blinks. Microsaccades were merged into one if they occurred less than 20ms from each other (Martinez-Conde et al., 2006). Attentional directions were gained by plotting detected microsaccades in a 2D space. Microsaccades in the direction of the side of the face represent attention toward the face.

### Design and Data Analysis

A mixed subjects’ design was employed. Avoidance was continuous independent variable, priming condition was grouping variable (neutral vs. secure), and Block (pre-priming vs. post-priming) and Facial expression (angry vs. neutral) were within participant variables. Dependent variables were Orientation (AOI/dot-probe), Microsaccadic Direction (% microsaccades away from the face), PANAS.

Data were analyzed with Linear Mixed Effects Models (Gallucci, 2019) using The Jamovi Project (2019). First, we ran four separate Linear Mixed Effects Models analyses on the dependent variables. To build the fixed effects structure, we built each model bottom up, adding one main effect/interaction at a time, using log likelihood ratio tests, Akaike information criterion (AIC) and Bayesian information criterion (BIC) to assess goodness of fit. We computed degrees of freedom using Satterthwaite approximation for each independent variable and interaction. Visual inspection of Q-Q plots did not reveal deviations of homoscedasticity or normality.

## Results

### Microsaccadic Eye Movement and Behavioral (RT) Data Convergence (Hypothesis 1)

The microsaccadic eye-movement attentional index correlated with the AOI from the dot-probe task (Pearson’s *r*=−0.202, *p*=0.010). As it reflected percentage of microsaccades away from the stimulus, while positive values on the AOI indicate vigilance, the negative correlation means that the higher % microsaccades away from the stimulus, the more the AOI denoted disengagement. See Supplementary Material 2.1 for descriptive statistics and exploratory correlation analyses, and Supplementary Material 2.2 for control analyses.

### Mixed Models

The best-fit model for Orientation (AOI) included Block (pre- vs. post-priming) and Avoidance. The overall model (fixed + random effects) captured 40.3% of the variance (Rm2=0.111, Rc2=0.403; AIC=−724.628, BIC=−679.916). The best-fit model for Microsaccadic Direction included Avoidance, Block, Condition, and the Condition × Avoidance interaction. The overall model (fixed + random effects) captured 40.2% of the variance (Rm2=0.050, Rc2=0.402; AIC=1273.650, BIC=1278.901). For all models, visual inspection of Q-Q plots did not reveal any obvious deviations of homoscedasticity or normality. See Supplementary Material 2.3 for model details.

#### High Avoidance Will Predict Lower Vigilance Toward Angry Faces Compared to Neutral Faces Pre-priming (Hypothesis 2)

**Figure 1:**
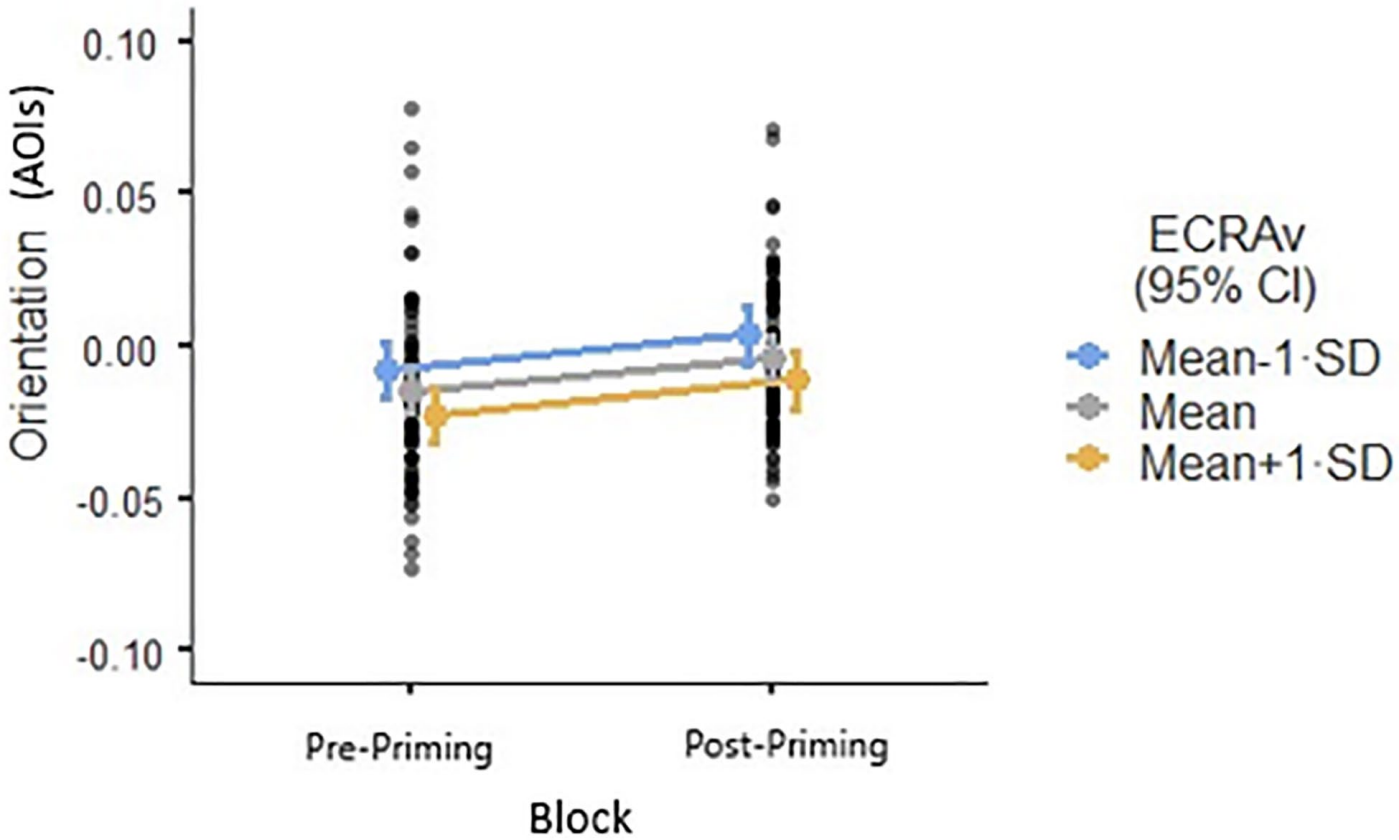
Attention to facial stimuli as a function of avoidance (ECR-R), as captured by the dot-probe task where orientation represents Attentional Orientation Indexes (AOIs). Y-axis values represent AOIs where a negative value indicates attentional disengagement, and a positive value indicates attentional engagement. A value of 0 indicates no attentional bias.

**Figure 2:**
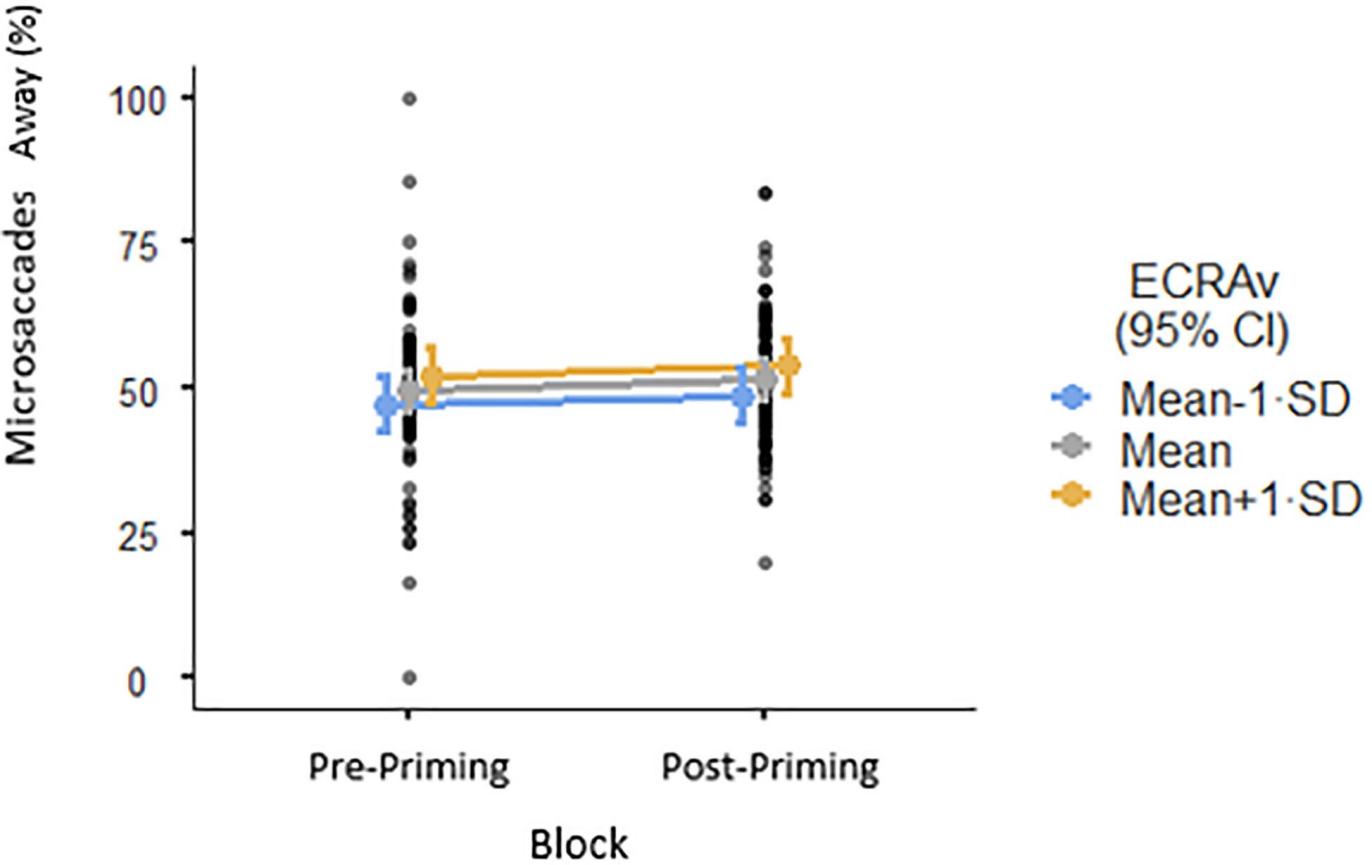
Attention to facial stimuli as a function of avoidance (ECR-R), as captured by microsaccades away from the stimuli.

In the model for Orientation, the interaction between Block, Avoidance, and Expression was not significant (*p*=0.591), suggesting that avoidance did not predict lower vigilance by type of facial expression post-priming. However, since Avoidance as main effect was significant (*β*=−0.007, *p*=0.016), the higher the individual’s avoidance score the more likely they were to attend away from facial stimuli both pre- and post-priming, suggesting lower vigilance to faces (Figure 1). In the model for Microsaccadic Direction, the interaction between Block, Avoidance, and Expression was not significant (*p*=0.918). No single variable reached significance in this model (Figure 2).

#### Secure Priming Will Have an Influence on Attention to Facial Expressions of Low Avoidance Participants but Not High Avoidance Participants (Hypothesis 3)

**Figure 3:**
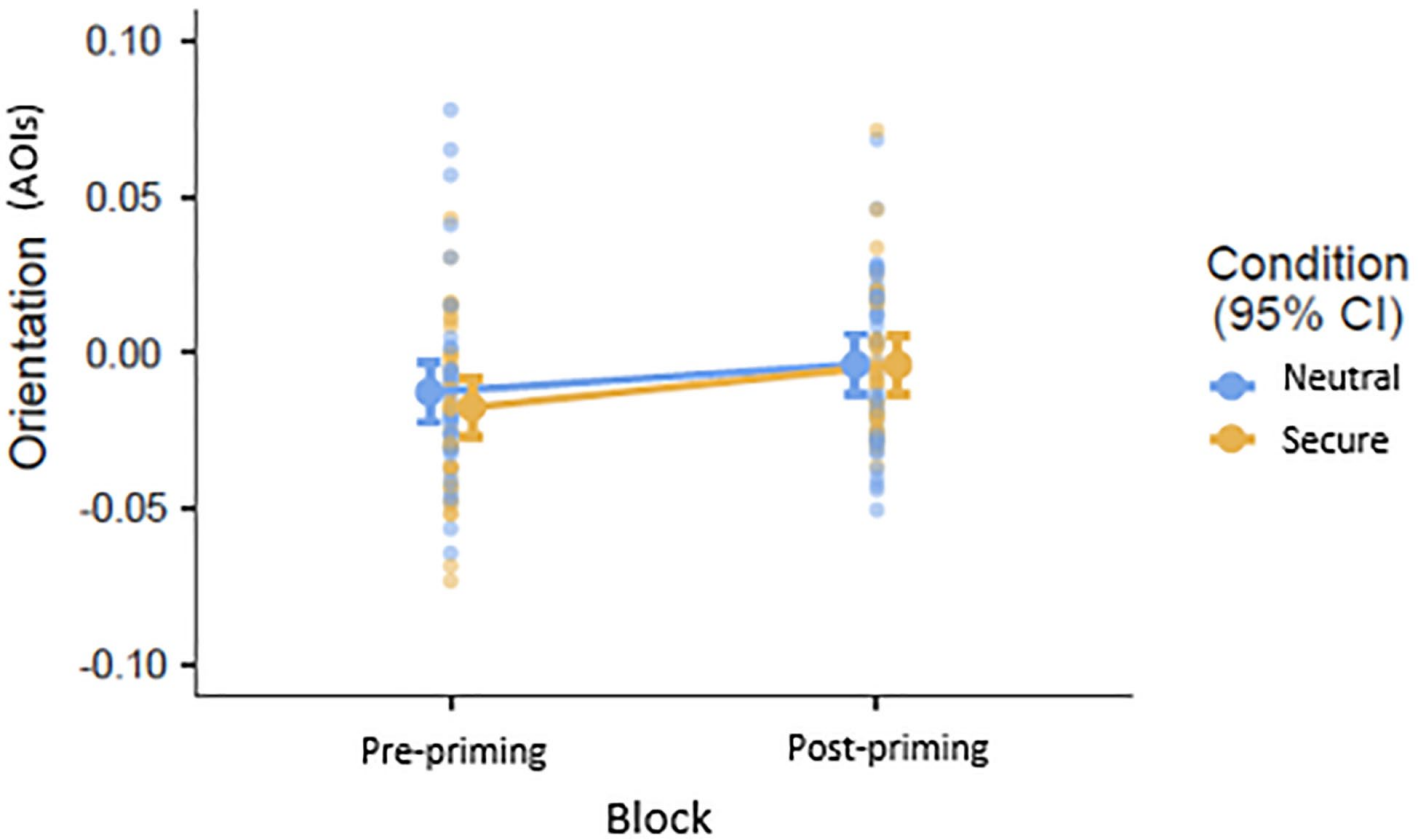
Attention pre- and post-priming: Increased vigilance to all facial stimuli as indicated by reaction times in the dot-probe task where orientation represents Attentional Orientation Indexes (AOIs). Y-axis values represent AOIs where a negative value indicates attentional disengagement, and a positive value indicates attentional engagement. A value of 0 indicates no attentional bias.

**Figure 4:**
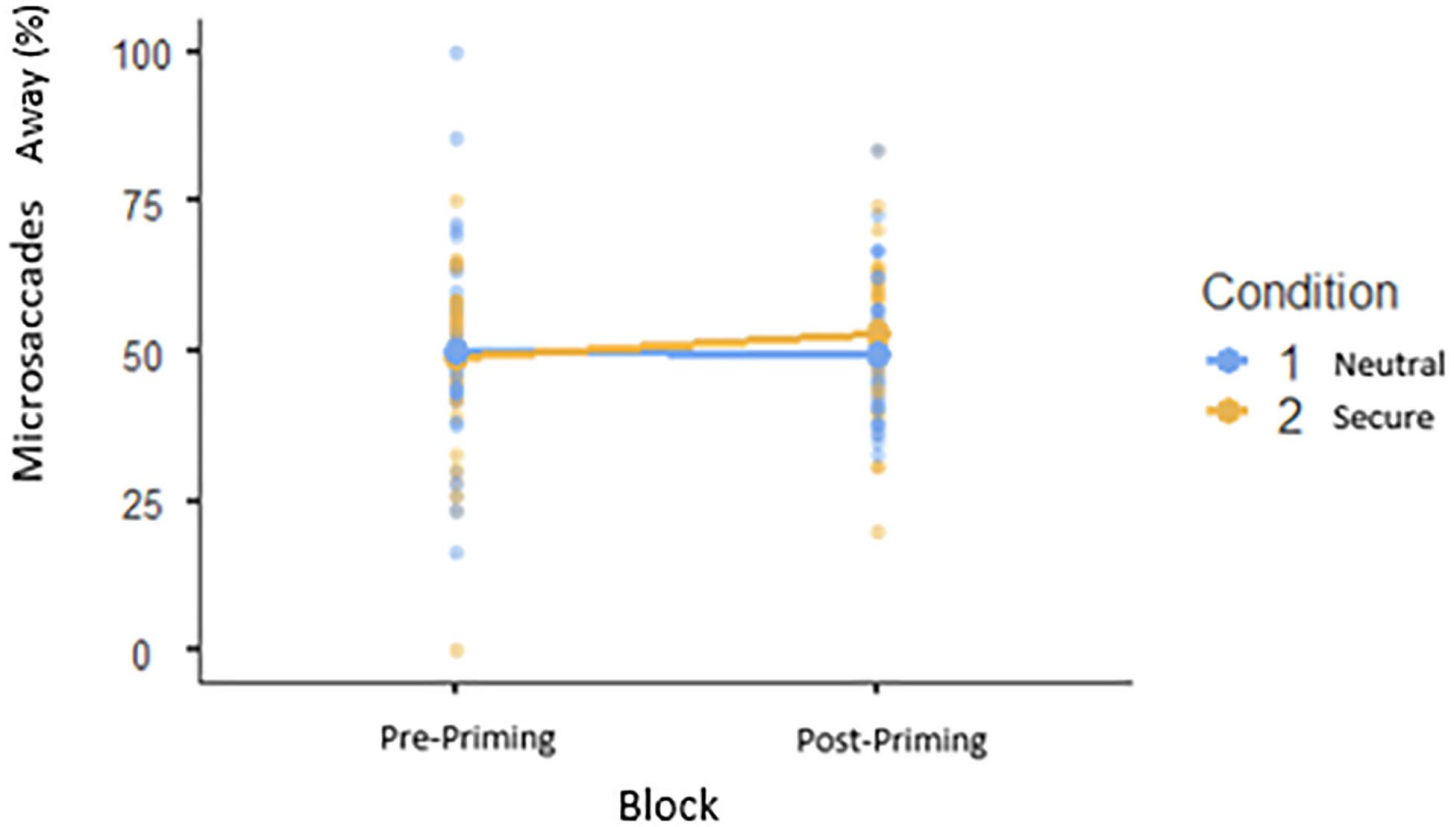
Attention pre- and post-priming: No difference captured by analysis of microsaccades away from the stimuli.

In the model for Orientation, the interaction between Block, Avoidance, and Condition was not significant (*p*=0.721), but a significant effect of Block (*β*=0.011, *p*=0.001) suggests that participants in both conditions showed increased vigilance to facial stimuli (both expressions) post-priming (Figure 3). The difference at baseline between participants in the secure priming and control conditions was not significant (*p*=0.402). In the model for Microsaccadic Direction, the interaction between Block, Avoidance, and Condition was not significant (*p*=0.735), nor were the main effects of Block (*p*=0.261) or Condition (*p*=0.600; Figure 4).

## Discussion

We assessed the impact of attachment avoidance and security priming on attentional biases toward emotional faces. Providing partial support for our hypotheses, the dot-probe data revealed that levels of avoidance were linked to attentional disengagement. However, the effect was found for all faces, regardless of emotional value. Microsaccades correlated with the RT-data, suggesting that they captured attention similarly, suggesting that this approach may be appropriate for the study of early attentional biases associated with attachment avoidance. Surprisingly, there was no effect of security priming on attention.

### Attachment Avoidance and Security Priming

High avoidance did not predict lower vigilance toward angry compared to neutral faces pre-priming. However, attention orientations in the dot-probe task did show less vigilance for facial stimuli in participants with higher avoidance scores, for both angry and neutral faces, in both priming conditions and across blocks (pre- and post-priming). Microsaccadic orientation did not significantly capture these findings.

Contrary to expectations, microsaccadic direction was not affected by security priming. The attention orientation from the dot-probe task showed increased vigilance to faces with time, but regardless of priming condition, level of avoidance or emotional valance of the face. Thus, participants became faster over time in congruent trials. In line with evidence of enhanced perspective taking and empathy from both writing (Manney, 2008; Oatley and Djikic, 2008) and reading stories (Djikic et al., 2013; Oatley, 2016), it is possible that felt security may have been temporarily increased by the mere act of story creation, regardless of condition/content of the story. Indeed, although neutral stories did not contain secure-base script interactions, some did include both another person and an interaction.

Specifically for avoidant individuals, poorer memory for attachment-related events in general (Fraley et al., 2000a), and positive attachment events in specific (Vrticka et al., 2008; Vrticka and Vuilleumier, 2012; Winer and Salem, 2016), can have made it more difficult to create stories that included secure-base interactions. Indeed, the amount scripted secure-base knowledge in participants’ stories was inversely related to attachment avoidance, indicating that participants high in attachment avoidance were less strongly primed. While a review of security priming research recommends subliminal priming for avoidant individuals so as to bypass avoidant defenses (Gillath and Karantzas, 2019), the emotional salience of such priming is unclear (Mayer and Merckelbach, 1999; Andriopoulos and Kafetsios, 2015). The priming procedure used here was arguably more emotionally salient than a subliminal prime, but not explicitly personal. It appears nevertheless that the procedure may have not bypassed avoidant regulation strategies, consistent with previous studies (Andriopoulos and Kafetsios, 2015). Because non-personal narrative tasks have not been used as security priming tasks before, more research is necessary for clarifying their usefulness, particularly concerning bypassing avoidant disengagement strategies.

### Microsaccades and the Attentional Orientation Index

Our microsaccadic eye-movement measure correlated strongly with the dot-probe attentional behavior measures, providing some first evidence of microsaccadic eye movements as a sophisticated methodology for assessing avoidant attentional biases. Behavioral data from the dot-probe task have previously produced mixed results (Fox et al., 2001; Price et al., 2015). Eye tracking to increase the dot-probe task validity tracked overt eye movements by instructing participants to fixate their eyes (Petrova et al., 2013), assuming that data after removing trials where overt eye movements occurred capture covert attention allocation. Removing this assumption, our measure of microsaccadic movements comprises explicit information on attention allocation at the point of stimulus presentation, proving a useful complement, or replacement, of dot-probe behavioral data as early attention allocation indexes.

Finally, the accurate detection of microsaccades is technically complex. The large individual variation in number of microsaccadic eye movements in our data (10 to 369 microsaccades over trials) could reflect differences in the physiological modulation of microsaccade rate generation. For instance, there is evidence that rates of microsaccadic eye movements are strongly associated with heartbeat (Ohl et al., 2016). However, given the early days in microsaccadic eye-movement detection, it cannot be excluded that available tools and algorithms may not optimally resolve the difficulties involved in detecting and measuring these eye movements (Nyström et al., 2016; Poletti and Rucci, 2016; Hooge et al., 2019). Additionally, future research could analyze, in addition to direction, where exactly in the visual field microsaccades occurred.

### Limitations

Notwithstanding methodological novelty and stringent controls, the study suffers limitations. Although compensated by the use of repeated measures, our sample size was relatively small. As the present study was the first to incorporate analysis of microsaccadic movement data in a dot-probe design for attentional biases, it was not feasible to work with a sample larger than 40 participants. In fact, experiments employing such eye-tracking procedures are typically based on no more than 20 participants. Thus, it was not possible to analyze both attachment avoidance and attachment anxiety biases with acceptable statistical power. Nor could we perform *a-priori* sample calculations. Given the strength of association between microsaccades and RTs in our data, future studies should aim at 45–50 participants. Notably, the significant association between RTs from the dot-probe task and our attentional measure based on microsaccades constitutes evidence of validity of the dot-probe task in the study of attentional biases, but future research should continue to assess its psychometric properties to this end.

In the present study, we compared angry and neutral facial expressions. Previous studies have used contemptuous faces (Chun et al., 2015), which convey negative interpersonal information, such as social rejection and criticism (Fischer and Roseman, 2007), or positive faces (Dewitte and De Houwer, 2008; Bantin et al., 2016; Winer and Salem, 2016) that convey positive interpersonal information. The inclusion of additional facial expressions would have made possible more informative comparisons. Furthermore, temporal effects of the microsaccadic eye movements were not investigated here. Future research ought to undertake an analysis of the temporal nature of the microsaccadic movements in the stimulus presentation window. Such analysis may reveal with more accuracy the timing of the disengagement stage of visual behavior.

Finally, the fact that participants high in attachment avoidance were less strongly primed limits our capacity to draw conclusions on effects of security priming on avoidant attentional biases. Future research should focus on further exploration of suitable priming methods to bypass avoidant deactivation, as this remains a challenge.

## Conclusion

Attachment avoidance in the present study was associated with deactivated attention from all facial stimuli. This avoidant strategy was robust, expressing itself independently of attachment security priming. Reaction time data and microsaccadic eye movements strongly correlated, suggesting that they capture attention similarly. Therefore, microsaccadic analysis could be useful to capture avoidant attentional strategies. While analysis of microsaccades appears to be promising for studying early patterns of attention, the large variability within the data suggest caution and larger sample sizes is advisable for establishing more robust evidence of the usability and validity of the method.

## Data Availability Statement

The original contributions presented in the study are included in the article/Supplementary Material, further inquiries can be directed to the corresponding author.

## Ethics Statement

The studies involving human participants were reviewed and approved by Lund University Department of Psychology Ethics Committee. The patients/participants provided their written informed consent to participate in this study.

## Author Contributions

RM: conceptualization, methodology, investigation, data curation, formal analysis, writing – original draft, writing – review and editing, and visualization. EP: conceptualization, methodology, data curation, formal analysis, writing – original draft, writing – review and editing, supervision, project administration, and funding acquisition. All authors contributed to the article and approved the submitted version.

## Funding

The study was partly funded by the Swedish Research Council (2019–02787) and the Crafoord Foundation (2019–1024), both awarded to EP and by Lund University Department of Psychology Research Funding. The funders had no role in study design, data collection and analysis, decision to publish, or preparation of the manuscript.

## Conflict of Interest

The authors declare that the research was conducted in the absence of any commercial or financial relationships that could be construed as a potential conflict of interest.

## Publisher’s Note

All claims expressed in this article are solely those of the authors and do not necessarily represent those of their affiliated organizations, or those of the publisher, the editors and the reviewers. Any product that may be evaluated in this article, or claim that may be made by its manufacturer, is not guaranteed or endorsed by the publisher.
