# Supplementary material for: The Study of Security Priming on Avoidant Attentional Biases: Combining Microsaccadic Eye-Movement Measurement With a Dot-Probe Task
Source: Front Psychol. 2021 Oct 21;12:726817. doi: 10.3389/fpsyg.2021.726817 (PMC8566336; doi:10.3389/fpsyg.2021.726817)
Supplement: Supplementary file 2 [file Data_Sheet_2.PDF]

1 Materials

1.1 Secure-Base and Neutral priming and scoring

Table S1 presents the word-prompts for two story titles (‘Math test’ involving a troublesome exam, and ‘Accident’ involving a cycling accident) in the security priming condition, taken from the Secure Base Script Test (SBST: Psouni & Apetroaia, 2014), and two (‘Trip to the park’ and ‘An afternoon shopping’) in the neutral priming condition, inspired from stories in the Attachment Script Assessment (ASA; Waters and Rodrigues-Doolabh, 2004). While the SBST was developed originally for assessment of attachment scripts in children and adolescents, the ASA was originated in the context of assessment of mothers’ attachment scripts. We have observed that the SBST assesses attachment scriptedness reliably, independently of whether the participant has experience of parental caregiving or not. In the present study, SBST-scriptedness was (negatively) correlated to ECR-R Avoidance and Anxiety scores.

Table S1.

Word prompts in the four story-titles. Secure priming condition story titles were, ‘Math Test’ and ‘Accident’. Neutral priming condition story titles were, ‘Trip to the Park’ and ‘An Afternoon Shopping’.

**Math Test (Psouni & Apetroaia, 2014)**		
Robin (Emma)	sad	study plan
math-test	mother/father	together
unprepared	talk	feel better
many mistakes	help	bedtime
**Accident (Psouni & Apetroaia, 2014)**		
Robin (Emma)	sad	stitches
new bike	mother/father	hug
fast	talk	bike repair
accident	help	watch film
**Trip to the Park**		
Alex	pavement	bench
bike	bottle	sit
park	trash can	wall
Fence	walk	gate
**An Afternoon Shopping**		
Alex	shop	sit
car	browse	stairwell
mall	buy	walk
parking	errands	exit

Transcribed stories were coded following a coding manual (Psouni & Apetroaia, 2013). A score “7” indicates descriptions of interpersonal interactions characterized by sensitivity and responsiveness to psychological states, while scores of “6” and “5” indicate lesser degree of these features. Stories with minimum evidence of secure-base knowledge are scored as “4”. Stories focused on actions or events, with no mention of emotional states or interactions, are scored as “3”. The score “2” denotes very limited descriptions of interactions from only one character’s perspective. Distinctively odd-content stories receive a score of “1”.

1.2 Attentional Orientation Task

Figure S1.

Illustration of a trial in the dot-probe task showing either an angry (left) face, or a neutral (right) face.

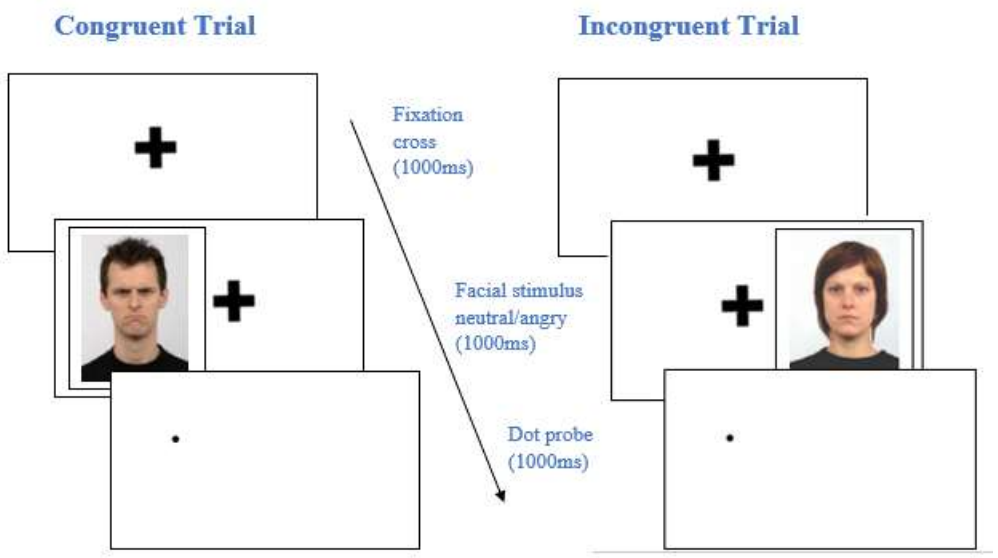

2 Results

2.1 Descriptive statistics

Attachment avoidance and anxiety were both negatively correlated to strength of scripted secure-base knowledge in participants' stories (SBST-score) (Table S2). Trait and state anxiety correlated positively with attachment anxiety (and avoidance) and negative affect, and negatively with positive affect (Table S3). The secure priming and neutral priming groups did not differ regarding age, gender, relationship status, state and trait anxiety, or negative affect before priming (Wilcox and t-tests) but differed on positive affect before priming (Neutral condition M = 36.2, Secure condition M = 30.7, $p < .001$).

Table S2.

Descriptive statistics for participant age, attachment anxiety/avoidance (ECR-R), attachment scripts (SBST), as well as state/trait anxiety (STAI) and affect (PANAS) (N = 40)

Variable	*M*	Median	*SD*	Minimum	Maximum
Age	27.40	26.00	4.42	21.00	39.00
Attachment Anxiety (screening ECR-R)	2.93	3.03	0.71	1.22	3.94
Attachment Avoidance (screening ECR-R)	2.89	2.75	1.04	1.22	5.72
Mean SBST	3.56	3.50	1.17	1.00	6.50
State Anxiety (STAI)	36.70	35.00	11.00	21.00	71.00
Trait Anxiety (STAI)	40.10	38.00	11.10	22.00	67.00
Pre-Priming Positive Affect (PANAS)	33.50	33.50	6.29	18.00	49.00
Post-Priming Positive Affect (PANAS)	32.70	32.00	7.41	19.00	50.00
Pre-Priming Negative Affect (PANAS)	14.30	13.00	4.69	10.00	30.00
Post-Priming Negative Affect (PANAS)	13.70	12.00	4.84	10.00	31.00

Not. Mean SBST, based on data from the Secure Priming group (n = 20), indicates amount of attachment security reflected in participants' stories.

Table S3.

Correlations among variables (N = 40)

	1	2	3	4	5	6	7	8
1 ECR-R Anxiety	—							
2 ECR-R Avoidance	.412*	—						
3 Mean SBST	-.230	-.250*	—					
4 State anxiety	.366*	.172*	.097	—				
5 Trait anxiety	.415*	.212*	.098	.739*	—			
6 Negative Affect	.272*	.203*	.077	.633*	.500*	—		
7 Positive Affect	-.090	.054	.070	-.414	-.407*	-.306*	—	
8 Age	-.092	.054	.212	.164*	.190*	.220*	-.040	—

* *p < .05, two-tailed.*

Mean RTs in the dot-probe task by condition (secure priming vs. neutral priming) and block (pre-priming vs. post-priming) appear in Table S4. AOIs for both neutral and angry faces were negative values indicating attentional disengagement from faces (M = -.0151 pre-priming, and M = -.0039 post-priming). Microsaccadic Direction followed the same pattern, with more microsaccades directed away from the facial stimulus (53.75% in both pre- and post-priming blocks). See Table S5 for descriptive statistics for microsaccade rate data. Table S6 presents Pearson's correlations of all these attention indicating variables and attachment avoidance.

Table S4.

Mean RTs (ms) pre- and post-priming (dot-probe task)

Facial Expression	Secure Priming condition (n = 20)				Neutral Priming condition (n = 20)			
	Pre-Priming		Post-Priming		Pre-Priming		Post-Priming	
	M	*SD*	*M*	*SD*	*M*	*SD*	*M*	*SD*
Angry	391.55	99.04	369.50	98.32	406.96	101.85	372.54	114.74
Neutral	393.25	97.77	371.88	110.26	410.58	110.01	375.87	120.00

Table S5.

Descriptives for microsaccade data, where M = Mean rate of occurrence

	Block					
Facial Expression	Pre-Priming			Post-Priming		
	M	*Total*	*SD*	*M*	*Total*	*SD*
Angry	49.88	1995	40.97	49.00	1960	45.43
Neutral	50.73	2029	40.82	50.13	2005	45.91

Table S6.

Correlations (Pearson's) between Attachment Avoidance (ECR) and attentional measures: dot probe data (RTs and AOIs) and microsaccades away from the stimulus

	Pre-priming Neutral	Pre-priming Angry	Post-priming Neutral	Post-priming Angry
1 RTs Incongruent	-0.152	-0.147	-0.147	-0.155
2 RTs Congruent	-0.040	-0.023	-0.060	-0.023
3 AOIs	-0.250	-0.273	-0.263	-0.318*
4 MS Away	0.189	0.123	0.270	0.193

* *p < .05.*

2.2 Control analyses, Affect

Since participants in the neutral priming condition reported higher positive affect pre-priming than those in the secure priming condition (*p* = .001), Condition as main effect predicted positive affect ($F_{(1, 38)}$ = 5.63, *p* = .023). However, a significant Condition X Block interaction ($F_{(1, 118)}$ = 4.03, *p* = .047) indicated that only positive affect of participants neutrally primed decreased post-priming. The best-fit model for Positive Affect included Block (pre- vs. post-priming), Condition, and their interaction. The model (fixed + random effects) captured 40.3% of variance (Rm2=.114, Rc2=.817; AIC = 924.791, BIC = 936.346), see Tables S5 for details.

Table S7.
Mixed Linear Model parameter estimates for positive affect

Fixed Effects Parameter Estimates				95 % Confidence Interval				
Names	Effect	Estimate	SE	Lower	Upper	df	*t*	*p*
(Intercept)	(Intercept)	33.075	.959	31.1955	34.954	38.0	34.49	<.001
Condition	2 – 1	-4.550	1.918	-8.3090	-.791	38.0	-2.37	.023
Block	2 – 1	-.750	.473	-1.6777	.178	118.0	-1.58	.116
Condition * Block	2 – 1 * 2 – 1	1.900	.947	.0446	3.755	118.0	2.01	.047

For negative affect there was no significant interaction between Condition and Block (*p* = .775). The best-fit model included only Block (pre- vs. post-priming), $F_{(1, 119)}$ = 2.54, β = -.625, *p* = .114. The overall model (fixed + random effects) captured 73.4% of the variance (Rm2=.004, Rc2=.734; AIC = 850.357, BIC = 861.642).

2.3 Mixed Linear Models for Orientation and Microsaccades

Table S8.
Fixed Effect Omnibus tests regarding Attentional Orientation (Dot-Probe)

	F	*Num df*	*Den df*	*p*
Block	10.89	1	119.0	.001
ECRAv	6.33	1	38.0	.016

Table S9.
Model parameter estimates regarding Attentional Orientation (Dot-Probe)

Fixed Effects Parameter Estimates				95 % Confidence Interval				
Names	Effect	Estimate	SE	Lower	Upper	df	*t*	*p*
(Intercept)	(Intercept)	-.00948	.00292	-.01521	-.00375	38.0	-3.24	.002
Block	2 – 1	.01120	.00340	.00455	.01786	119.0	3.30	.001
ECRAv	ECRAv	-.00706	.00281	-.01256	-.00156	38.0	-2.52	.016

Table S10.
Fixed Effect Omnibus tests regarding Microsaccadic eye movements

Fixed Effect Omnibus tests	*F*	*Num df*	*Den df*	*p*
ECRAv	2.284	1	36.0	0.139
Block	1.290	1	119.0	0.258
Condition	0.280	1	36.0	0.600
ECRAv ✻ Condition	0.658	1	36.0	0.423

Table S11.
Model parameter estimates regarding Microsaccadic eye movements

Fixed Effects Parameter Estimates				95 % Confidence Interval				
**Names**	**Effect**	**Estimate**	**SE**	**Lower**	**Upper**	**df**	***t***	***p***
(Intercept)	(Intercept)	50.37	1.57	47.294	53.45	36.0	32.096	<.001
ECRAv	ECRAv	2.29	1.52	-0.681	5.27	36.0	1.511	0.139
Block	2 – 1	1.95	1.71	-1.412	5.30	119.0	1.136	0.258
Condition	2 – 1	1.66	3.14	-4.490	7.81	36.0	0.529	0.600
ECRAv * Condition	ECRAv * 2 – 1	-2.46	3.03	-8.410	3.49	36.0	-0.811	0.423
